# Optimizing CD8^+^ T cell-based immunotherapy via metabolic interventions: a comprehensive review of intrinsic and extrinsic modulators

**DOI:** 10.1186/s40164-024-00575-7

**Published:** 2024-10-22

**Authors:** Zihao Zhou, Jiarong Zheng, Ye Lu, Zizhao Mai, Yunfan Lin, Pei Lin, Yucheng Zheng, Xu Chen, Rongwei Xu, Xinyuan Zhao, Li Cui

**Affiliations:** 1 Stomatological Hospital, School of Stomatology, Southern Medical University, Guangzhou, 510280 Guangdong China; 2 Department of Dentistry, The First Affiliated Hospital, Sun Yat-Sen University, Guangzhou, 510080 China; 3 School of Dentistry, University of California, Los Angeles, Los Angeles, 90095 CA USA

**Keywords:** CD8^+^ T cells, Metabolic modulation, T cell-based immunotherapy, Exogenous metabolites, Therapeutic optimization

## Abstract

CD8^+^ T cells are integral to the effective management of cancer and infectious diseases due to their cytotoxic functions. The efficacy of these cells is profoundly influenced by their metabolic state, which regulates their activation, differentiation, and longevity. Accordingly, the modulation of metabolic pathways within CD8^+^ T cells is crucial for enhancing the effectiveness of T cell-based immunotherapy. Precise metabolic control is paramount in optimizing therapeutic outcomes and minimizing potential toxicities associated with treatment. Importantly, the potential of exogenous metabolites to augment CD8^+^ T cell responses is critically evaluated, especially through in vivo evidence that underscores their therapeutic promise. This review also addresses current challenges, including the need for precise control of metabolic modulation to avoid adverse effects, the development of targeted delivery systems to ensure efficient metabolite delivery to CD8^+^ T cells, and the inherent variability of metabolic states among patients that may influence treatment outcomes. Addressing these hurdles will be crucial for the successful integration of metabolic interventions into established immunotherapeutic regimens.

## Background

CD8^+^ T cells play a central role in adaptive immunity, critical for combating both infectious diseases and cancer due to their cytotoxic capabilities. These cells target and eliminate cells infected by pathogens or transformed into cancer cells, making them essential for a wide range of immunotherapies [1–3]. Additionally, CD8^+^ T cells are vital in vaccination strategies, enhancing the strength and longevity of immune responses, which is key for effective disease prevention [4–6]. Advancements in understanding CD8^+^ T cell regulation have led to innovative treatments for autoimmune diseases, where adjusting their activity helps mitigate tissue damage caused by improper immune responses. Therefore, the strategic manipulation of CD8^+^ T cells represent a fundamental therapeutic approach that spans infectious disease control, cancer treatment, and autoimmunity, underscoring their indispensable role in advancing immunotherapy and vaccine development [7–9].

Normal metabolism is fundamental for the proper function of CD8^+^ T cells, impacting their ability to respond effectively during immunotherapy. The metabolic health of these cells dictates their proliferation, differentiation, and effector functions [10, 11]. However, the challenging conditions within tumor microenvironment (TME) or sites of infection, such as hypoxia and nutrient scarcity, can disrupt these metabolic pathways, leading to suboptimal T cell function. This metabolic disruption impairs the ability of CD8^+^ T cells to effectively eradicate cancer cells or control infections, undermining the success of immunotherapeutic interventions [12–14]. Interestingly, fatty acid synthesis (FAS) is essential for T cell activation and antitumor function, particularly in contexts where lipid synthesis supports membrane formation and signaling pathways [15]. However, the solid TME impairs CD8^+^ T cell metabolism by inducing acetyl-coenzyme A carboxylase activity, promoting lipid biogenesis over fatty acid oxidation (FAO), which can hinder the bioenergetics and antitumor function of tumor-infiltrating T cells (TILs) [16]. This underscores the complexity of the TME's influence on metabolic pathways, where both FAS and FAO may play context-dependent roles in shaping T cell responses.

To counteract these adverse conditions, one strategic approach in immunotherapy involves the exogenous addition of metabolic substrates to modify the local environment and restore or enhance T cell metabolic functions. This strategy not only enhances the immediate effectiveness of treatments like checkpoint inhibitors and adoptive T cell transfers but also contributes to the long-term success of immune responses by supporting the maintenance of a robust population of memory T cells [17–21]. Therefore, enhancing the metabolic flexibility of CD8^+^ T cells can help them overcome the metabolic blockades imposed by harsh conditions, thereby sustaining their activity and efficacy in hostile environments.

In this review, we provide a detailed examination of the metabolic regulation of CD8^+^ T cells, emphasizing its pivotal role in enhancing their functional efficacy. This regulation is crucial, as metabolic processes directly influence the activation, differentiation, and longevity of CD8^+^ T cells in various immunotherapeutic contexts. Importantly, the review pioneers a critical analysis of using exogenous metabolites to augment CD8^+^ T cell-based immunotherapies. It specifically highlights the in vivo evidence that supports the therapeutic potential of these metabolites, distinguishing this approach as a novel enhancement strategy. Additionally, the review addresses the prevailing challenges in this research area and offers a forward-looking perspective on the potential developments and innovations that may overcome current limitations, thereby advancing the field of CD8^+^ T cell-based immunotherapy.

## Overview of CD8^+^ T cells

**Fig. 1:**
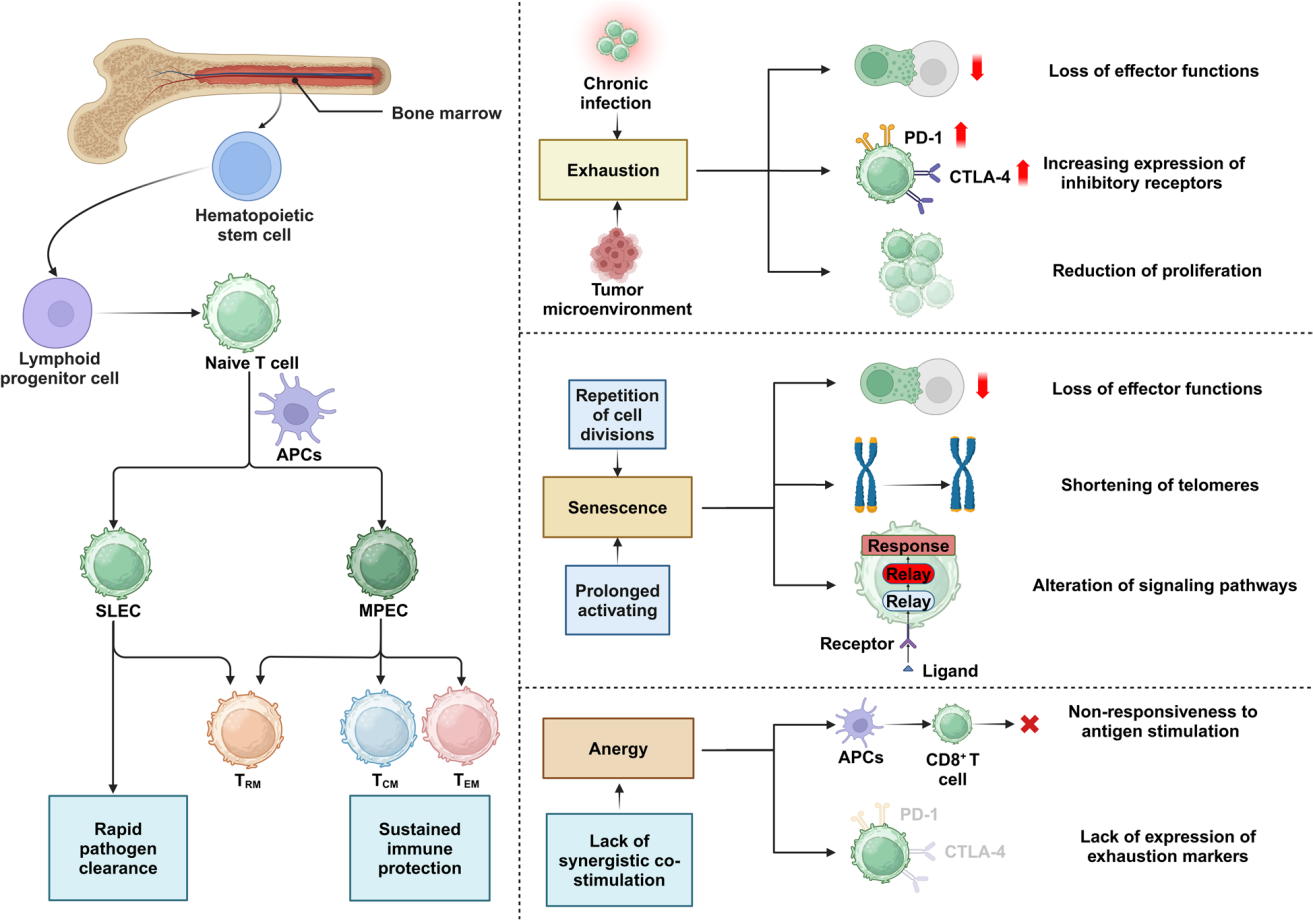
CD8^+^ T cell development, differentiation, and the impacts of tumor microenvironments and chronic infections on their function. Hematopoietic stem cells in the bone marrow give rise to naive T cells, which interact with APCs and differentiate into either short-lived effector cells (SLEC) or memory precursor effector cells (MPEC), leading to central memory (T_CM_), effector memory (T_EM_), or tissue-resident memory (T_RM_) cells. Chronic infections and tumors induce CD8^+^ T cell exhaustion, characterized by increased inhibitory receptors (PD-1, CTLA-4, etc.), loss of effector functions, and reduced proliferation. Repeated cell divisions and prolonged activation cause senescence, marked by telomere shortening and altered signaling pathways. Lacking adequate co-stimulation results in anergy, where cells become unresponsive to antigens. Created in BioRender.com

CD8^+^ T cells, originating from common lymphoid progenitors in the bone marrow, undergo rigorous maturation in the thymus to ensure their ability to recognize antigens presented by major histocompatibility complex class I (MHC class I) molecules [22]. These cells start as naive T cells, patrolling the body to detect antigens associated with infected or transformed cells. Upon encountering their specific antigen, they activate and differentiate into effector subtypes such as short-lived effector cells (SLECs) and memory precursor effector cells (MPECs). SLECs provide rapid pathogen clearance, while MPECs develop into long-lived memory T cells, crucial for sustained immune protection (Fig. 1) [23, 24]. The memory compartment of CD8^+^ T cells is further categorized into several subtypes, including central memory T (T_CM_) cells and effector memory T (T_EM_) cells [25]. T_CM_ cells are known for their longevity and high proliferative potential upon re-exposure to antigen, making them crucial for long-term immunity. In contrast, T_EM_ cells provide immediate protective functions upon re-encountering antigens [26]. Notably, T_EM_ and T_CM_ exhibit distinct metabolic and functional profiles influenced by T cell receptor (TCR), CD28, and co-stimulatory signals from chimeric antigen receptor (CAR) constructs, leading to rapid IFN-γ production driven by glycolysis, while 4-1BB domains in CARs promote central memory traits through FAO and mitochondrial biogenesis [27, 28]. In adoptive cell therapies, modifications such as Rapamycin treatment or Wnt-β-catenin signaling activation (e.g., with TWS119) enhance memory T cell survival, repopulation capacity, and anti-tumor efficacy. Specifically, Rapamycin-treated T cells demonstrate improved functionality and longevity in transplant patients, while TWS119 induction favors the accumulation of stem cell-like memory T cells, linked to increased survival mediated by the NF-κB pathway [29–31]. Transcription factor 1 (TCF-1) modulation also plays a crucial role, enhancing memory CD8^+^ T cell proliferation and response to infection without similarly benefiting CD4^+^ T cells [32]. These strategies underscore the importance of targeting specific signaling pathways and metabolic programs to refine T cell-based therapies for more effective cancer treatment and immunotherapy. Additionally, tissue-resident memory T (T_RM_) cells represent a specialized subset residing permanently within tissues, ready to respond swiftly to previously encountered pathogens or tumor cells [33, 34]. For instance, T_RM_ cells in the skin continuously patrol among keratinocytes, detecting antigen-expressing cells within minutes to hours post-infection. This rapid surveillance by T_RM_ cells is crucial for controlling secondary infections at previously infected sites [35].

However, in chronic infection or cancer settings, CD8^+^ T cells often face a harsh microenvironment or persistent antigen exposure that can lead to T cell exhaustion. This state is characterized by a loss of effector functions, increased expression of inhibitory receptors such as programmed death receptor 1 (PD-1) and cytotoxic T-lymphocyte-associated protein 4 (CTLA-4), and metabolic impairments [36, 37]. Exhausted T cells show a reduced ability to proliferate and secrete cytokines, which significantly impairs their ability to control infections and tumors [38–40]. Notably, T cell senescence, on the other hand, refers to the state where T cells exhibit reduced effector functions, shortened telomeres, and altered signaling pathways due to repeated cell divisions and prolonged activation [41, 42]. Senescent T cells show impaired proliferative capacity, decreased cytokine production, and diminished cytotoxicity, which severely hampers their ability to combat infections and tumor cells effectively [43]. For instance, T cell senescence within TMEs, driven by both malignant and regulatory T cells (Tregs), limits the effectiveness of immunotherapies. Ataxia-telangiectasia mutated (ATM)-associated DNA damage and mitogen-activated protein kinase (MAPK) signaling are identified as key mediators of T cell aging. Inhibiting these pathways, in conjunction with anti-PD-L1 therapy, robustly enhances antitumor responses in mouse models of melanoma, lung, and breast cancer (BC), supporting a dual-target strategy against T cell senescence and exhaustion to potentiate cancer immunotherapy [44]. In addition to exhaustion and senescence, anergy, a state of T cell non-responsiveness to antigen stimulation without the expression of overt exhaustion markers, also contributes to the functional impairment of CD8^+^ T cells [45]. Tumor-antigen-specific CD4^+^ T cells exhibit early transient expansion but rapidly develop anergy in tumor-bearing hosts. This anergic state impairs their response to peptide antigens and prevents effective priming, posing a significant barrier to therapeutic vaccination and antitumor immunity [46]. The ability to harness and manipulate these distinct functional states and trafficking patterns of CD8^+^ T cells holds significant promise for targeted therapies, particularly in cancer and chronic infections, where directing these cells to effectively recognize and eliminate target cells can dramatically improve therapeutic outcomes. Understanding the nuanced roles and origins of these subsets is crucial in advancing CD8^+^ T cell-based therapies, emphasizing the need for precise control over their development and function in clinical settings.

Notably, CD8^+^ T cells are pivotal in mediating cytotoxic responses against tumors, yet the critical support provided by CD4^+^ T cells is essential for optimizing these responses. CD4^+^ T cells enhance the efficacy of CD8^+^ T cells by supplying essential cytokines such as interleukin-2 (IL-2), which boosts CD8^+^ T cell proliferation and survival, and by differentiating into Th1 cells that produce IFN-γ, further amplifying CD8^+^ T cell activation within TME [47]. In the context of immunotherapy, robust CD4^+^ T cells significantly enhance responses to checkpoint inhibitors and augment the outcomes of adoptive T cell therapies. For instance, increased IL-5 production from CD4^+^ T cells has been shown to drive systemic eosinophil expansion and enhance eosinophil infiltration in tumors. This elevation in eosinophils, further supported by IL-33, is crucial for activating CD8^+^ T cells within TME, thus amplifying the therapeutic efficacy of immune checkpoint blockade (ICB) [48]. In addition, in adoptive cell therapy for lung cancer (LC), miR-7 deficiency in CD4^+^ T cells enhances Th1 polarization and activation, markedly reducing tumor growth and boosting CD8^+^ T cell responses. This effect is mediated through upregulation of MAPK4 and altered signaling in key pathways, highlighting miR-7's potential as a target to amplify CD4^+^ T cell-mediated antitumor efficacy [49]. Thus, while CD8^+^ T cells target and eliminate tumor cells, the collaborative dynamics with CD4^+^ T cells are indispensable, underscoring the need for therapeutic strategies that harness the synergistic potential of both T cell subsets to maximize cancer immunotherapy efficacy.

## Metabolic regulation of CD8^+^ T cell functionality

**Fig. 2:**
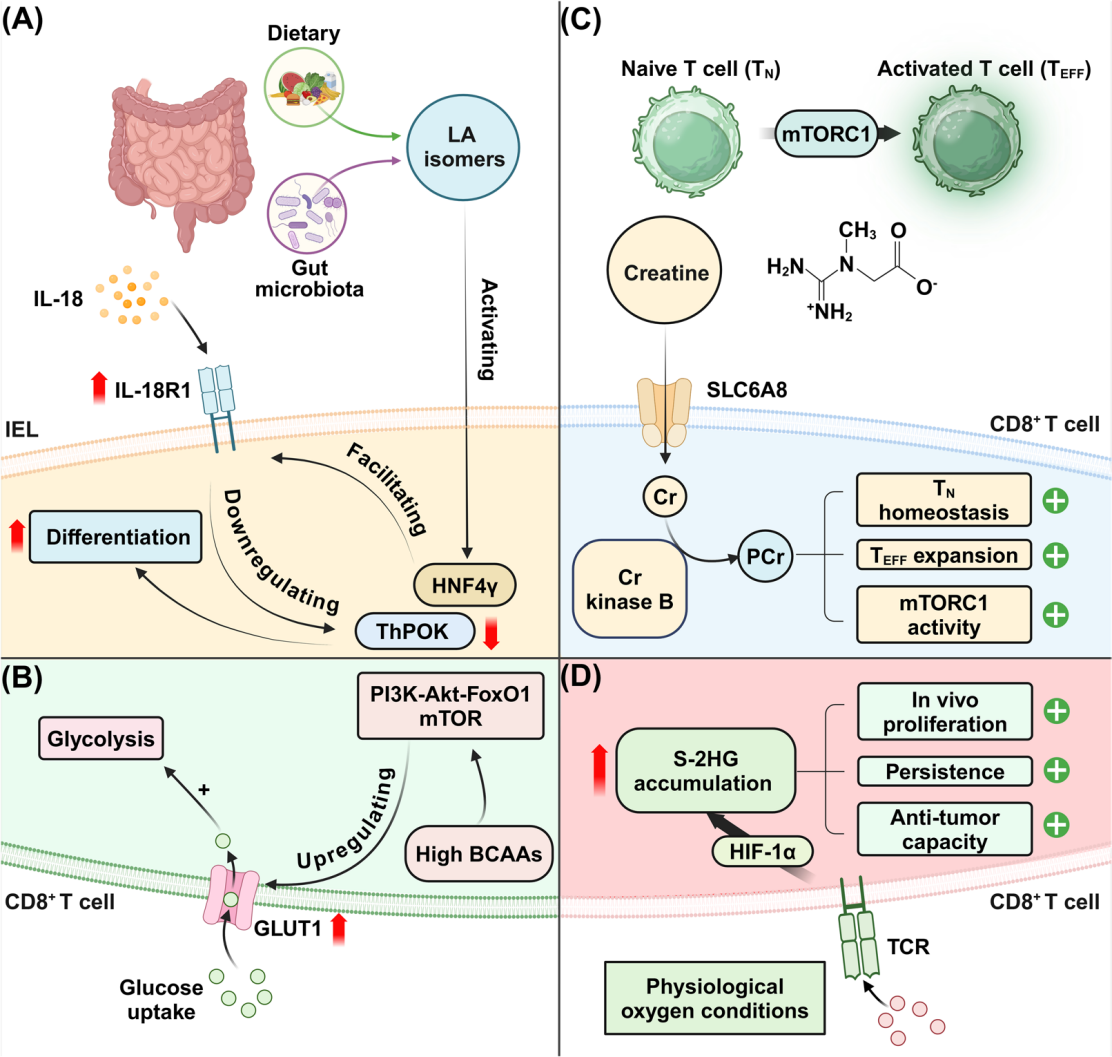
Metabolic regulation of CD8^+^ T cell functionality. **A** Dietary and gut microbiota-derived LA isomers activate HNF4γ, which facilitates IL-18R1 expression, downregulating ThPOK while enhancing CD8^+^ T cell differentiation through IL-18 signaling. **B** High branched-chain amino acids (BCAAs) upregulate the PI3K-Akt-FoxO1-mTOR pathway, enhancing glucose uptake through GLUT1 and glycolysis in CD8^+^ T cells, thereby supporting their energy production and function. (C) Creatine, transported into cells via SLC6A8, is phosphorylated by creatine kinase B, producing phosphocreatine (PCr). PCr supports the homeostasis of naive T cells (T_N_), the expansion of activated effector T cells (T_EFF_), and increases mTORC1 activity, ultimately enhancing CD8^+^ T cell proliferation and anti-tumor responses. (D) Under physiological oxygen conditions, the accumulation of S-2-hydroxyglutarate (S-2-HG) is mediated by HIF-1α, this process promotes CD8^+^ T cell proliferation, persistence, and anti-tumor capacity. Created in BioRender.com

Metabolites play a pivotal role in regulating the functionality of CD8^+^ T cells, serving as crucial modulators of their metabolic, proliferative, and cytotoxic activities (Fig. 2). These small molecules, derived from cellular metabolic processes or external sources, influence CD8^+^ T cell responses through various biochemical pathways [50, 51]. For instance, low apolipoprotein A1 (ApoA1) levels are linked to impaired CD8^+^ T cell function in endometrial, ovarian, and LCs. ApoA1 promotes antitumor activity of CD8^+^ T cells by stabilizing HIF-1α, which regulates glycolysis. This pathway enhances CD8^+^ T cell infiltration and function, driving tumor necrosis [52]. Additionally, CD8^+^ TILs in clear cell renal cell carcinoma exhibit impaired mitochondrial metabolism, characterized by hyperpolarized, fragmented mitochondria and reactive oxygen species (ROS). This metabolic dysfunction, linked to downregulated superoxide dismutase 2 (SOD2), disrupts glucose uptake and glycolysis, compromising TIL function [53]. Notably, in severe COVID-19, CD8^+^ T cells show mitochondrial dysfunction due to galectin-3 suppressing NRF1 translocation, reducing mitochondrial complex III/IV gene expression. Galectin-3 inhibition with TD-139 restores mitochondrial biogenesis, highlighting the role of mitochondrial metabolism in CD8^+^ T cell dysfunction [54]. In addition, ROS regulate CD8^+^ T cell metabolic fitness in the TME by triggering SUMO-specific protease 7 (SENP7)-mediated deSUMOylation of phosphatase and tensin homolog (PTEN), promoting PTEN degradation and preventing metabolic defects. SENP7-deficient CD8^+^ T cells show reduced glycolysis and OXPHOS, impairing proliferation and antitumor function [55].

Interestingly, dietary and microbial factors converge to modulate gut linoleic acid (LA) isomers, such as conjugated LAs, which in turn influence the mucosal immune system by regulating CD4^+^CD8αα^+^ intraepithelial lymphocytes (IELs) in the small intestine. Disruption of fatty acid isomerization in gut bacteria reduces these IELs, while restoration of conjugated LAs promotes their development through IL-18 signaling [56]. Similarly, in the small intestine, T_RM_ cells, particularly IELs, exhibit rapid metabolic adaptation to local availability of key nutrients like glucose, which critically governs their activation and functionality [57]. Interestingly, glutamine-derived polyamines and hypusine are crucial for CD8^+^ T cell metabolic reprogramming, influencing proliferation and the regulation of CD69 in T_RM_ CD8^+^ T cells. Inhibiting the glutamine/polyamine/hypusine pathway enhances the development and functional response of T_RM_ cells, increasing CD69 expression and cytokine production in CD8^+^ T cells [58]. Accumulation of branched-chain amino acids (BCAAs) enhances CD8^+^ T cell activity and antitumor immunity by upregulating Glut1 expression and boosting glucose metabolism. In *PP2Cm*-deficient mice, this results in increased glycolysis and oxidative phosphorylation (OXPHOS). BCAA supplementation mimics these effects and improves anti-PD-1 therapy efficacy, suggesting BCAAs as potential supplements to enhance CD8^+^ T cell function and immunotherapy outcomes [59].

Specific amino acids and lipids act as signaling molecules, activating pathways such as mammalian target of the rapamycin (mTOR), which is essential for CD8^+^ T cell growth and differentiation [60]. Creatine, a key circulating metabolite, plays a cell-intrinsic role in maintaining CD8^+^ T cell homeostasis and expansion. This function is mediated through creatine kinase B and the creatine transporter solute carrier family 6 member 8 (SLC6A8), which facilitate TCR-mediated activation of mechanistic target of rapamycin complex 1 (mTORC1) signaling essential for T cell expansion during infection [61]. Notably, metabolites can modulate the epigenetic landscape of these cells, influencing gene expression patterns crucial for their effector functions. Notably, enantiomers of the same metabolite, such as D-2-hydroxyglutarate (D-2-HG) and S-2-hydroxyglutarate (S-2-HG), can have distinct biological functions. D-2-HG accumulates in isocitrate dehydrogenase (IDH)-mutant acute myeloid leukemia (AML) and impairs dendritic cells (DC) differentiation, leading to a tolerogenic phenotype with reduced major histocompatibility complex class II (MHC class II) expression. This suppression limits the ability of T cells to recognize and lyse AML cells [62]. Gain-of-function IDH mutations produce D-2-HG, which impairs CD8^+^ T cell metabolism and antitumor activity by disrupting glycolysis and IFN-γ signaling via lactate dehydrogenase [63]. In contrast, S-2-HG accumulates in CD8^+^ T cells upon activation, mediated by HIF-1α under physiological oxygen conditions. This metabolite shifts the balance towards S-enantiomer dominance, enhancing CD8^+^ T cell differentiation through modifications in histone and DNA demethylation and stabilizing HIF-1α. These changes result in increased proliferation, persistence, and antitumor capacity of CD8^+^ T cells, positioning S-2-HG as a pivotal immune metabolite that connects metabolic and epigenetic changes to immune functionality [64].

## The advantages of using exogenous metabolites for enhancing CD8^+^ T cell-based immunotherapy

**Fig. 3:**
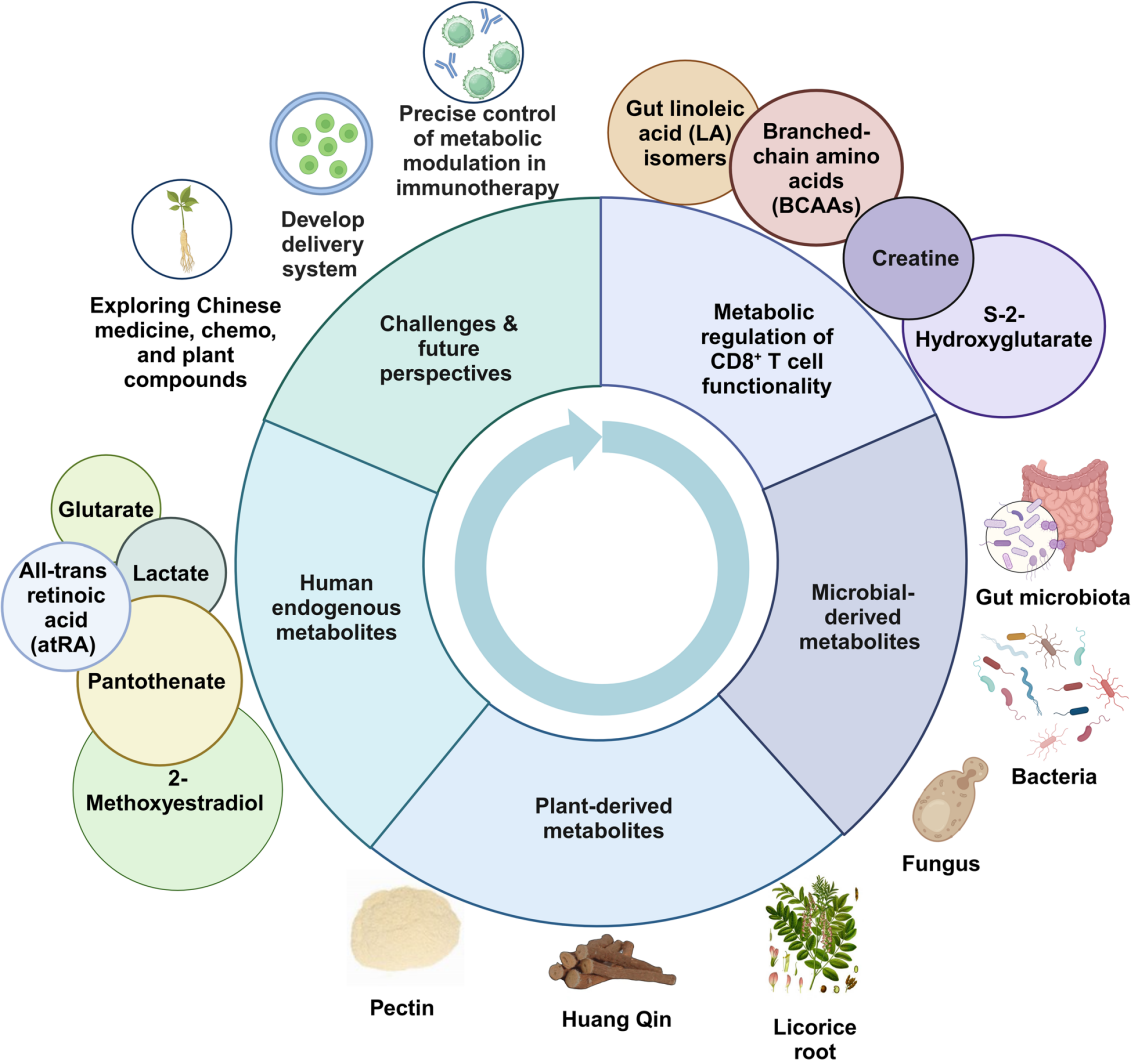
Enhancement of CD8^+^ T cell-based immunotherapy through the exogenous addition of various metabolites, supported by in vivo evidence. Gut linoleic acid isomers, branched-chain amino acids, creatine, and S-2-hydroxyglutarate modulate CD8^+^ T cell function. Microbial-derived metabolites from gut microbiota, including bacteria and fungi, significantly contribute to this modulation. Additionally, plant-derived compounds such as pectin, baicalin, and 18β-glycyrrhetinic acid, along with human endogenous metabolites like pantothenate and atRA, influence CD8^+^ T cell activity. This highlights the promising potential of precise metabolic modulation in enhancing CD8^+^ T cell function within TMEs or chronic infection contexts, addressing the challenges and future perspectives in improving CD8^+^ T cell-based immunotherapy. Created in BioRender.com

Enhancing CD8^+^ T cell-based therapies through the addition of exogenous metabolites has emerged as an advantageous strategy to optimize T cell function (Fig. 3) [18, 65–68]. The use of exogenous metabolites circumvents the potential risks associated with genetic modifications, such as insertional mutagenesis and the subsequent risk of oncogenesis. This approach ensures a safer therapeutic profile by avoiding the unintended genetic alterations that can arise from vector transduction. In addition, metabolic support via exogenous metabolites can help remodel the TME to be more supportive of immune activity [69, 70]. By providing essential nutrients, T cells can better compete with tumor cells and other suppressive elements in the TME, thus enhancing their antitumor efficacy [71]. Moreover, exogenous metabolites can enhance the metabolic flexibility and adaptability of CD8^+^ T cells. By supplementing key metabolic intermediates, T cells might switch more effectively and swiftly between glycolysis and OXPHOS, optimizing their energy production based on the environmental conditions, and thereby maintaining their functionality under stress [72, 73]. Furthermore, by optimizing the metabolic environment for T cells, exogenous metabolites can reduce the need for higher doses of chemotherapeutic agents, thereby minimizing their toxic side effects [74]. This can improve patient quality of life and reduce the overall treatment burden.

## Exogenous microbial-derived metabolites for enhancing CD8^+^ T cell-based immunotherapy

### Short-chain fatty acids (SCFAs)

**Fig. 4:**
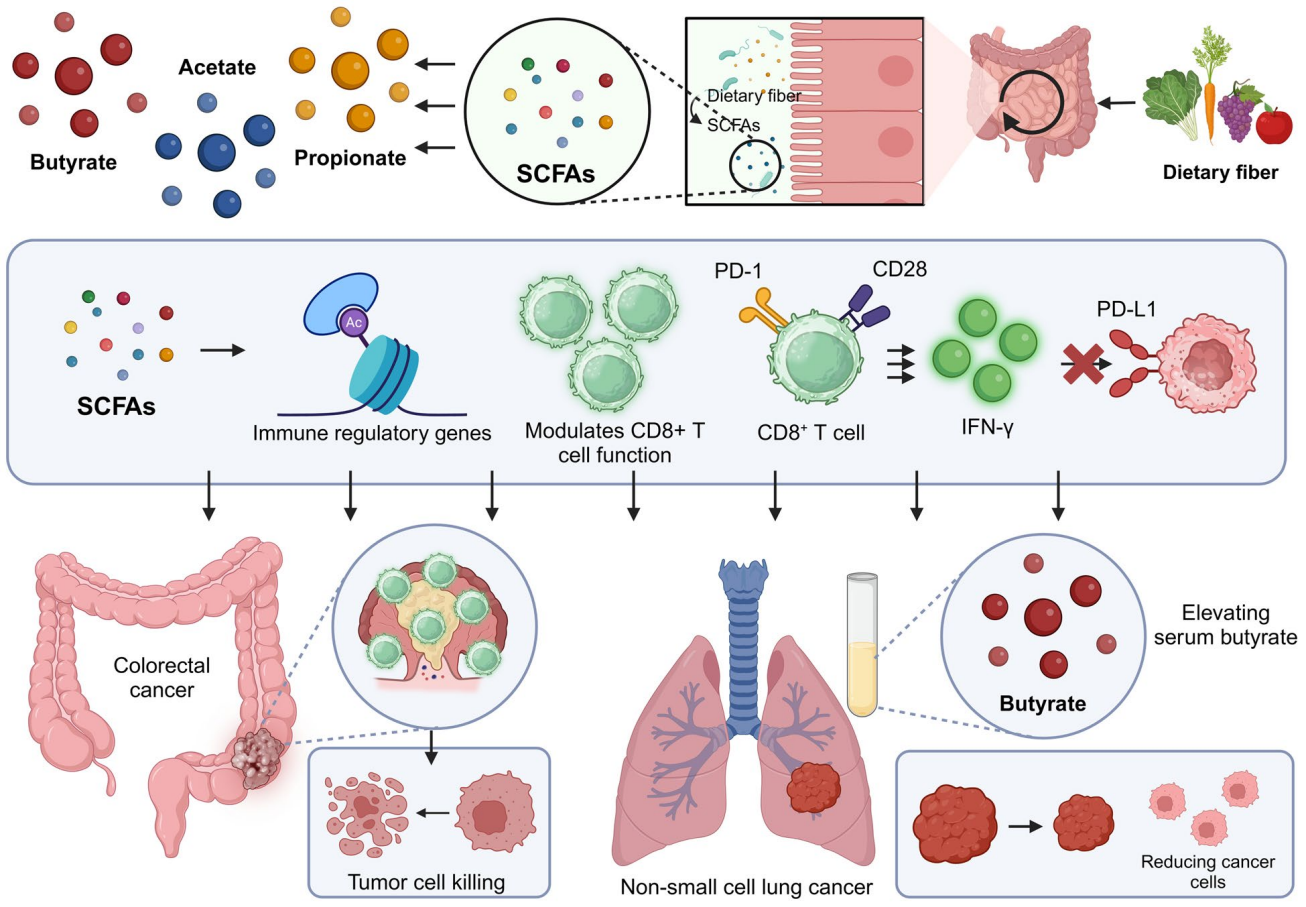
Enhancing CD8^+^ T cell immunotherapy through SCFA modulation. SCFAs, including acetate, propionate, and butyrate, are derived from dietary fiber fermentation by gut microbiota. These SCFAs modulate immune regulatory genes and enhance CD8^+^ T cell function by influencing PD-1 and CD28 pathways, leading to increased IFN-γ production and reduced PD-L1 expression. Elevated serum butyrate levels have been shown to reduce cancer cells in non-small cell lung cancer (NSCLC). Butyrate can enrich CD8^+^ T cells and enhance tumor cell killing in colorectal cancer (CRC). This highlights the role of SCFAs in improving anti-tumor immune responses, emphasizing the potential of dietary interventions to support CD8^+^ T cell-based immunotherapy. Created in BioRender.com

SCFAs are a group of metabolites produced by the microbial fermentation of dietary fibers in the gut. These compounds play a crucial role in modulating immune responses, particularly enhancing the functionality and efficacy of CD8^+^ T cells, key players in antiviral and anticancer immunity (Fig. 4; Table 1). For instance, SCFAs enhance CD8^+^ T cell activation in colorectal cancer (CRC), particularly in microsatellite instability CRCs, which are more sensitive to SCFA treatment than chromosomally unstable CRCs. SCFAs induce DNA damage in CRC cells, upregulating chemokine, MHC class I, and antigen presentation genes, thereby stimulating a positive feedback loop with activated CD8^+^ T cells [75]. In the high-fiber diet-fed mice model, SCFAs from the diet promoted the effector function of CD8^+^ T cells by enhancing cellular metabolism, serving as a substrate for FAO, and specifically interacting with free fatty acid receptor 3 (FFAR3), thereby boosting their intrinsic anti-viral responses and overall viral immunity [76]. Therefore, understanding the mechanisms through which microbial-derived SCFAs influence CD8^+^ T cells opens new avenues for therapeutic interventions aimed at enhancing CD8^+^ T cell efficacy across a spectrum of cancer and viral infections.

**Table 1:** Exogenous metabolites in disease: CD8^+^ T cell modulation

Metabolites	Associated disease	Mechanism of action	Specific CD8^+^ T cell effects	Clinical application	Refs.
Butyrate, Propionate	CRC	Induce DNA damage, upregulate chemokine, MHC class I	Enhance CD8^+^ T cell activation	Enhance CD8^+^ T cell efficacy in CRC immunotherapy	[75]
Butyrate	NSCLC	Increase histone acetylation at key immune regulatory genes	Enhance anti-PD-1 therapy by boosting PD-1 and CD28 expression	Biomarker for immunotherapy enhancement in NSCLC	[77]
Butyrate	CRC	Inhibit IFN-γ-induced PD-L1 upregulation through the acetylation of STAT1	Boost CD8^+^ T cell cytotoxicity and infiltration	Enhance anti-PD-L1 immunotherapy in CRC	[78]
Butyrate	CRC	Modulate CD8^+^ T cell function via the ID2-dependent IL-12 signaling pathway	Boost the antitumor responses	Adjunct therapy with oxaliplatin	[74]
Butyrate	-	Promote oxidative metabolism and long-term survival of CD8^+^ T cells	Enhance CD8^+^ T cell memory and recall responses	Boost immunotherapeutic outcomes	[79]
Butyrate	CRC	Activate CD8^+^ T cells via TLR5 binding and NF-κB signaling	Enhance anti-PD-1 immunotherapy efficacy	Potential therapeutic adjuvant in CRC treatment	[80]
Acetate	OPC	Activate GPR43, enhance IFN-γ and chemokine expression	Boost CD8^+^ T cell recruitment and antitumor activity	Therapeutic agent in OPC treatment	[81]
Acetate	BC	Increase CD8^+^ T cells and reverse stress-promoted cancer progression	Increase antitumor responses	Immunotherapy for BC in stressed/depressed patients	[82]
Butyrate	Type 1 diabetes	Mitigate cytotoxic activity of CD8^+^ T cells through anti-inflammatory effects	Reduce cytotoxic function of CD8^+^ T cells	Potential therapeutic role in autoimmune diabetes	[83]
Indole-3-lactic acid	CRC	Elevate IL-12A in dendritic cells, enhance H3K27 acetylation binding to enhancers	Improve tumor-infiltrating CD8^+^ T cells	Enhance CRC immunotherapy efficacy	[90]
Indole-3-aldehyde	Melanoma	Boost CD8^+^ T cell IFN-γ production via AhR in TME	Enhance effectiveness of ICIs	Tryptophan-rich diet as melanoma adjunct therapy	[91]
Indole-3-propionic acid	Melanoma, BC, CRC	Enhance CD8^+^ T cell stemness and foster progenitor exhausted T cells via increased H3K27 acetylation	Enhance αPD-1 immunotherapy	Enhance ICB therapies	[92]
Indole-3-carboxylic acid	CRC	Inhibit IDO, reduce kynurenine levels, and alleviate its inhibitory impact on effector T cells via AhR competition	Decrease Treg infiltration, enhance CD8^+^ T cell activity	Augment anti-PD1 therapies effectiveness in CRC	[93]
Tryptophan	Pancreatic ductal adenocarcinoma	Elevate AhR activity in TAMs; augment ICB therapy efficacy	Increase pro-inflammatory CD8^+^ T cells within tumors	Suppress tumor growth in PDAC	[95]
ITE	Autoimmune encephalomyelitis	Boost Treg and DC differentiation via AhR	Induce Treg differentiation	Potential immunotherapy agent in autoimmune disorders	[96]
Indole-3-aldehyde	AIH	Tet2-deficient, AhR signaling	Promote Tc1 differentiation	Potential therapeutic role in AIH	[97]
Urolithin A	Pancreatic cancer	Remodel TME, reduce stromal fibrosis, increase CD8^+^ T cells	Decrease PD-1 expression, improve T cell infiltration	Adjunct therapy with anti-PD-1 therapies	[101]
Urolithin A	Lymphoma, Melanoma	Activate ERK1/2, promote autophagy and metabolic boost via Urolithin A-ERK1/2-ULK1	Increase cellular metabolism, maintain optimal ROS levels	Boost CD8^+^ T cell function and persistence	[102]
Exopolysaccharide	Melanoma, CRC, BC	Induce CCR6^+^ CD8^+^ T cells in Peyer's patches, promote IFN-γ production	Boost CD8^+^ T cell count in CCL20-expressing tumors	Augment efficacy of anti-CTLA-4 and anti-PD-1 therapies	[103]
Ferrichrome	Pancreatic cancer	Reprogram TAMs; induces TLR4 pathway activation to suppress FPN1 expression	Elevate tumor infiltration, improve anti-PD-L1 therapy	Potential combination therapy with ICIs	[104]
TMAO	TNBC	Trigger pyroptosis via PERK, boost CD8^+^ T cell antitumor immunity	Enhance immunotherapy response	Augment immunotherapy efficacy in TNBC	[105]
Gallic acid	CRC	Disrupt Foxp3 in Tregs, inhibit STAT3 phosphorylation and USP21 expression	Weaken Tregs function, boost CD8^+^ T cell IFN-γ production	Enhance anti-PD-1 therapies	[106]
Ascomylactam C	LC, Melanoma	Boost ROS production, trigger ER stress, activate PERK/eIF2α/ATF4/CHOP pathway	Induce immunogenic cell death, enhance infiltration	Potential immunotherapy agent in LC and melanoma	[107]

Butyrate, a gut microbiota SCFA, enhances antitumor immunity by increasing histone acetylation at key immune regulatory genes, thereby boosting PD-1 and CD28 expression in CD8^+^ T cells. This modulation enhances the efficacy of anti-PD-1 therapy and promotes antitumor cytokine production through T-cell receptor signaling. Elevated serum levels of butyrate correlate with improved responses in non-small cell lung cancer (NSCLC) patients, positioning butyrate as a potential therapeutic biomarker for immunotherapy enhancement [77]. Similarly, butyrate enhances anti-PD-L1 immunotherapy in CRC by modulating immune responses. It inhibits IFN-γ-induced PD-L1 upregulation through the acetylation of STAT1, reducing its expression and nuclear translocation. This action boosts CD8^+^ T-cell cytotoxicity and infiltration, suppressing tumor growth. However, direct PD-L1 overexpression in CRC cells negates butyrate's beneficial effects, highlighting its role in immune-mediated therapy optimization [78]. Notably, butyrate enhances the efficacy of oxaliplatin in cancer therapy by modulating CD8^+^ T cell function via the ID2-dependent IL-12 signaling pathway. This effect was observed both in vitro and in vivo, correlating with increased serum butyrate levels in oxaliplatin-responsive patients. This highlights the potential of integrating exogenous butyrate into conventional cancer treatments to boost immunotherapeutic outcomes [74]. Moreover, butyrate enhances CD8^+^ T cell memory by promoting oxidative metabolism and long-term survival. In germ-free mice, antigen-activated CD8^+^ T cells failed to form memory cells due to transcriptional impairments. Butyrate uncouples the tricarboxylic acid cycle from glycolysis, favoring OXPHOS through glutamine and fatty acid catabolism, thereby optimizing recall responses [79]. Interestingly, *Roseburia intestinalis*, notably depleted in CRC patients, suppresses tumor growth and enhances anti-PD-1 immunotherapy efficacy through butyrate production. In CRC mouse models, *Roseburia intestinalis* or butyrate reduces tumorigenesis, restores gut barrier integrity, and activates cytotoxic CD8^+^ T cells via Toll-like receptor 5 (TLR5) binding and NF-κB signaling. This suggests potential therapeutic benefits of *Roseburia intestinalis* and its metabolite butyrate as adjuvants in CRC treatment strategies [80]. Remarkably, oral microbiota dysbiosis, characterized by decreased *Lactobacillus*, reduces CD8^+^ T cell infiltration and promotes oropharyngeal cancer (OPC) development. *Lactobacillus* supplementation restores CD8^+^ T cell levels and antitumor activity, mediated by its metabolite acetate. Acetate activates G-protein-coupled receptor 43 (GPR43), enhancing IFN-γ and chemokine expression to boost CD8^+^ T cell recruitment and inhibit OPC progression. This highlights *Lactobacillus* and acetate as potential therapeutic agents in OPC treatment [81]. Likewise, chronic stress correlates with decreased *Blautia* and its metabolite acetate in BC patients, particularly those with depression. These reductions are linked to lower CD8^+^ T cell tumor infiltration and higher metastasis risks. Supplementing with *Blautia* and acetate enhances antitumor immune responses, suggesting their potential in immunotherapy for managing BC progression in stressed and depressed female patients [82].

In addition to its role in cancer, butyrate has been shown to play critical roles in mediating CD8^+^ T cell-based immune responses in various diseases. For instance, in type 1 diabetes, initial activation of islet-specific CD8^+^ T cells occurs in pancreatic lymph nodes, not due to molecular mimicry with gut microbiota-derived peptides but through intrinsic pancreatic antigens. These activated T cells subsequently migrate to gut lymphoid tissues, where they enhance their cytotoxic functions via bystander activation. The anti-inflammatory metabolite butyrate, however, can mitigate this enhanced cytotoxic activity, suggesting a potential therapeutic role in modulating T cell responses related to autoimmune diabetes [83]. In addition, sodium butyrate (NaB) enhances survival and mitigates sepsis-associated lung injury in mice by modulating immune responses and barrier functions. NaB administration reduces inflammation, as evidenced by decreased proinflammatory cytokines and biomarkers, while increasing anti-inflammatory cytokines and amphiregulin. It also improves tissue integrity and increases the proportion of Tregs, highlighting its therapeutic potential in enhancing gut-lung crosstalk and immunoregulation [84]. Moreover, butyrate and, to a lesser extent, propionate enhance CD8^+^ T cell function by increasing IFN-γ and granzyme B expression and shifting type 17 cytotoxic T lymphocytes (Tc17) towards a cytotoxic phenotype. These effects occur independently of SCFA receptors G-protein-coupled receptor 41 (GPR41) and GPR43, primarily through histone deacetylase inhibition. Additionally, higher acetate levels boost IFN-γ production via metabolic modulation and mTOR activation. These findings highlight the potential of SCFAs in augmenting CD8^+^ T cell-based immunotherapy for cancer and viral infections [85].

### Indole derivatives

**Fig. 5:**
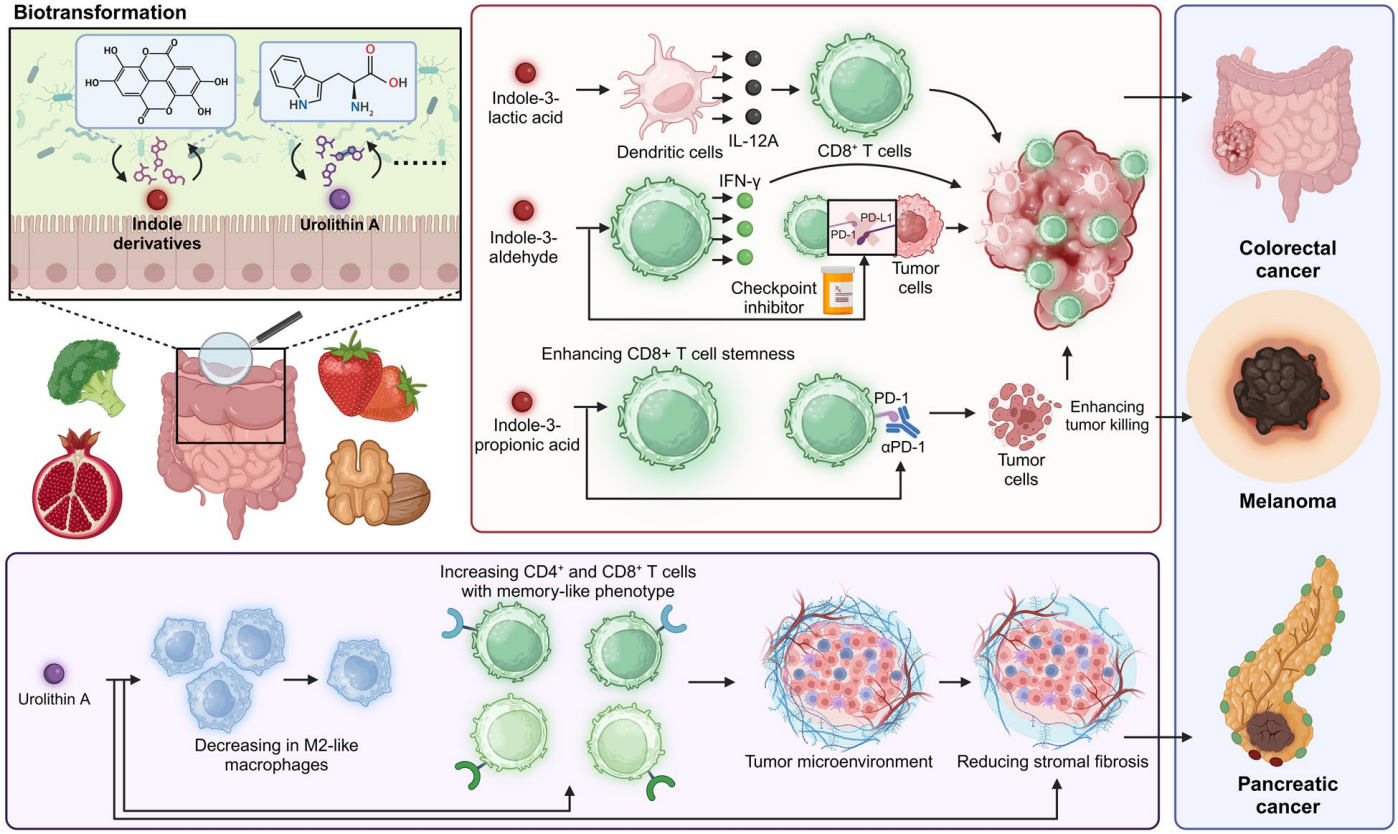
Bacterial-transformed metabolites enhance CD8^+^ T cell-based immunotherapy. Indole derivatives and urolithin A, produced through biotransformation of dietary components, enhance CD8^+^ T cell stemness and function. Indole-3-lactic acid and indole-3-aldehyde stimulate dendritic cells to produce IL-12A, promoting CD8^+^ T cell activation and IFN-γ production, which enhances tumor cell killing. Indole-3-propionic acid further supports this effect by reducing PD-1 expression on CD8^+^ T cells. Urolithin A decreases M2-like macrophages and increases CD4^+^ and CD8^+^ T cells with a memory-like phenotype, improving TME conditions and reducing stromal fibrosis. These mechanisms collectively enhance anti-tumor responses in colorectal cancer (CRC), melanoma, and pancreatic cancer, highlighting the therapeutic potential of bacterial-transformed metabolites in immunotherapy. Created in BioRender.com

Indole and its derivatives modulate CD8^+^ T cells by acting as aryl hydrocarbon receptor (AhR) ligands, promoting glycolysis and FAO, inhibiting the immunosuppressive indoleamine 2,3-dioxygenase (IDO) pathway, and enhancing cytokine production like IFN-γ and IL-12, thus supporting their cytotoxic function and immune responses [86, 87]. Indole derivatives such as indole-3-aldehyde (I3A) and indole-3-lactic acid are microbial metabolites produced from the fermentation of tryptophan by gut microbiota. These compounds significantly modulate immune responses, particularly influencing CD8^+^ T cell functionality. They operate through various mechanisms that are essential for maintaining immune homeostasis and enhancing anti-inflammatory pathways (Fig. 5) [88, 89]. For instance, *Lactobacillus plantarum L168* and its metabolite, indole-3-lactic acid, mitigate CRC by modulating immune response. Indole-3-lactic acid boosts IL-12A production in dendritic cells, enhancing histone 3 lysine 27 (H3K27) acetylation binding to its enhancer regions, which primes CD8^+^ T cell immunity. Additionally, it represses serum amyloid protein A 3 (SAA3)) expression, improving cholesterol metabolism and the efficacy of TILs [90]. In addition, probiotic *Lactobacillus reuteri* enhances antitumor immunity in melanoma through its metabolite, I3A, which promotes IFN-γ production in CD8^+^ T cells within the TME. This action bolsters the effectiveness of immune checkpoint inhibitors (ICIs). Essential to this process, AhR signaling in CD8^+^ T cells mediate the antitumor effects of I3A, with a tryptophan-rich diet further augmenting this response [91]. Moreover, *Lactobacillus johnsonii* and *Clostridium sporogenes* synergize to produce the metabolite indole-3-propionic acid, enhancing CD8^+^ T cell efficacy in ICB across multiple cancers. Indole-3-propionic acid modulates CD8^+^ T cell stemness and fosters progenitor exhausted T cells through increased H3K27 acetylation, boosting αPD-1 immunotherapy responsiveness [92]. Remarkably, *Lactobacillus gallinarum* enhances the efficacy of anti-PD1 therapy in CRC by modulating the immune landscape, particularly reducing Treg infiltration and boosting CD8^+^ T cell function. Its metabolite, indole-3-carboxylic acid, inhibits IDO, decreasing kynurenine production and disrupting kynurenine's suppressive effects on effector T cells by competing with the AhR. This metabolic interference significantly augments anti-PD1 therapy effectiveness [93]. In addition, L- and D-kynurenine induce T cell apoptosis by increasing β-oxidation and depleting fatty acids in CD4^+^ T cells, impairing their function. Replenishing fatty acids restores T cell viability, highlighting kynurenine's role in metabolic regulation. Dietary D-kynurenine achieves tissue concentrations similar to those in human cancers but induces only limited immunosuppression. These findings suggest that sub-suppressive kynurenine levels in human tumors may reduce the efficacy of IDO inhibition in clinical trials [94].

Importantly, the role of indole derivatives in modulating immune responses seems to be contradictory across different types of cancer. For instance, in pancreatic ductal adenocarcinoma, tumor-associated macrophages (TAMs) with high AhR activity, driven by *Lactobacillus*-mediated metabolization of dietary tryptophan to indoles, support tumor growth and suppress anti-tumor immunity. Inhibiting AhR or dietary manipulation of tryptophan and its metabolites decreases TAM immunosuppression, enhancing the efficacy of ICB and increasing the presence of pro-inflammatory CD8^+^ T cells within tumors [95].

Similarly, the influence of indole derivatives on immune response regulation appears to be intricate and contingent upon the specific autoimmune disorders. For instance, indole derivatives, such as the AHR ligand 2-(1′H-indole-3′-carbonyl)-thiazole-4-carboxylic acid methyl ester (ITE), play a crucial role in promoting immune tolerance by inducing Tregs. ITE acts on both T cells and dendritic cells, fostering a tolerogenic environment that supports Treg differentiation. This mechanism, involving retinoic acid signaling, has shown potential in suppressing autoimmune diseases like experimental autoimmune encephalomyelitis, highlighting indole derivatives as promising agents for treating autoimmune disorders [96]. Conversely, I3A, an AhR ligand produced by gut bacteria like *Lactobacillus reuteri*, drives autoimmune hepatitis (AIH) by promoting IFN-γ-producing type 1 cytotoxic T lymphocyte (Tc1) cell differentiation. In a Tet2-deficient model, AIH-like pathology is microbiota-dependent and triggered by hepatic translocation of I3A. Both IFN-γ and AhR signaling are critical, as blocking either pathway mitigates disease [97].

The contrasting roles of indole derivatives across different cancer contexts and autoimmune disorders suggest that their impact on immune modulation is highly nuanced and influenced by specific environments. Importantly, the therapeutic manipulation of microbial metabolites requires careful consideration of the systemic effects of these compounds. Given their capacity to modulate distant immune responses, strategies that target or mimic these metabolites need to be optimized based on individual patient microbiota profiles and cancer types to prevent unintended consequences such as enhanced tumor protection or immune suppression.

### Urolithin A

Urolithin A is a microbial-derived metabolite produced by the gut microbiota through the biotransformation of ellagitannins, which are polyphenolic compounds found in various fruits and nuts, such as pomegranates, strawberries, and walnuts [98]. This metabolite has garnered attention for its potential to modulate immune responses, particularly influencing CD8^+^ T cell functionality. Urolithin A has been shown to exert anti-inflammatory effects and enhance mitochondrial function in cells, which are crucial for the energy demands of CD8^+^ T cells during their activation and proliferation [99, 100]. The understanding of how Urolithin A affects CD8^+^ T cell dynamics offers promising avenues for dietary interventions aimed at optimizing immune health and function. For instance, Urolithin A enhances immunotherapy efficacy in pancreatic cancer by remodeling the TME. It reduces stromal fibrosis and M2-like macrophages while increasing CD4^+^ and CD8^+^ T cells with a memory-like phenotype and decreasing PD-1 expression. When combined with anti-PD-1 therapy, urolithin A significantly improves T cell infiltration and overall survival in a mouse model of pancreatic cancer, suggesting its potential as an adjunct therapy [101]. Similarly, urolithin A boosts CD8^+^ T cell antitumor efficacy by directly targeting ERK1/2 kinases, enhancing their activation and promoting autophagy and metabolic enhancement via the Urolithin A-ERK1/2-ULK1 pathway. This interaction increases cellular metabolism and maintains optimal ROS levels, thus improving CD8^+^ T cell function and persistence in tumor immunotherapy [102].

### Other microbial-derived metabolites

In addition to the microbial-derived metabolites mentioned above, other exogenous microbial-derived metabolites have also been shown to be crucial for enhancing CD8^+^ T cell-based immunotherapy. Dietary intake of microbial exopolysaccharide from *Lactobacillus delbrueckii subsp. bulgaricus* enhances anti-tumor immunity by inducing CCR6^+^ CD8^+^ T cells in both conventional and germ-free mice. These T cells, activated in Peyer's patches, increase in number within C–C motif chemokine ligand 20 (CCL20)-expressing tumors, promoting IFN-γ production and augmenting the efficacy of anti-CTLA-4 and anti-PD-1 therapies. Exopolysaccharide acts through a mechanism involving its phosphorylated structure and interaction with a lysophosphatidic acid receptor on CD8^+^ T cells [103]. Notably, a siderophore, ferrichrome, derived from *Lactobacillus casei*, reprograms TAMs and enhances CD8^+^ T cell infiltration in pancreatic cancer, reducing tumor burden. Ferrichrome activates the Toll-like receptor 4 (TLR4) pathway, suppressing ferroportin expression in macrophages, thereby facilitating an improved response to anti-PD-L1 therapy. This highlights a novel probiotic-derived intervention for boosting the efficacy of ICIs in cancer treatment [104]. In triple-negative breast cancer (TNBC), trimethylamine N-oxide (TMAO), a metabolite linked to commensal microbiota, correlates with an activated immune microenvironment and improved immunotherapy responses. TMAO facilitates tumor cell pyroptosis via the PERK pathway, thereby enhancing CD8^+^ T cell-mediated antitumor immunity. These insights highlight the potential of microbial metabolites like TMAO to augment immunotherapy efficacy in TNBC [105]. Moreover, gallic acid, a gut microbial metabolite, destabilizes Foxp3 in Treg cells and enhances ICB efficacy by inhibiting USP21 gene transcription and STAT3 phosphorylation. In colorectal cancer models, combined treatment with gallic acid and anti-PD-1 antibody reduces Treg cell function, boosts CD8^+^ T cell IFN-γ production, and restricts tumor growth, suggesting a potent therapeutic strategy to improve ICB outcomes by inducing T-helper-1-like Treg cells [106].

Interestingly, metabolites derived from fungi have been shown to significantly modulate immune responses. Ascomylactam C, a macrocyclic alkaloid from *Didymella sp.*, inhibits tumor growth and induces apoptosis in LC and melanoma cell models. In vivo, Ascomylactam C enhances CD4^+^ and CD8^+^ T cell infiltration in tumor tissues. Mechanistically, it boosts ROS production, triggers endoplasmic reticulum (ER) stress, and activates the PERK/eIF2α/ATF4/CHOP pathway, leading to immunogenic cell death [107]. Notably, a novel dibenzofuran derivative, isomycousnine enamine (iME), isolated from the endophytic fungus *Mycosphaerella nawae*, selectively suppresses CD4^+^ T-cell activation and proliferation while affecting overall T-cell immune responses. This selective immunosuppressive effect on CD4^+^ T cells by iME highlights its potential as a targeted immunosuppressant, offering a promising avenue for immunotherapy regulation [108].

## Exogenous plant-derived metabolites for enhancing CD8^+^ T cell-based immunotherapy

**Fig. 6:**
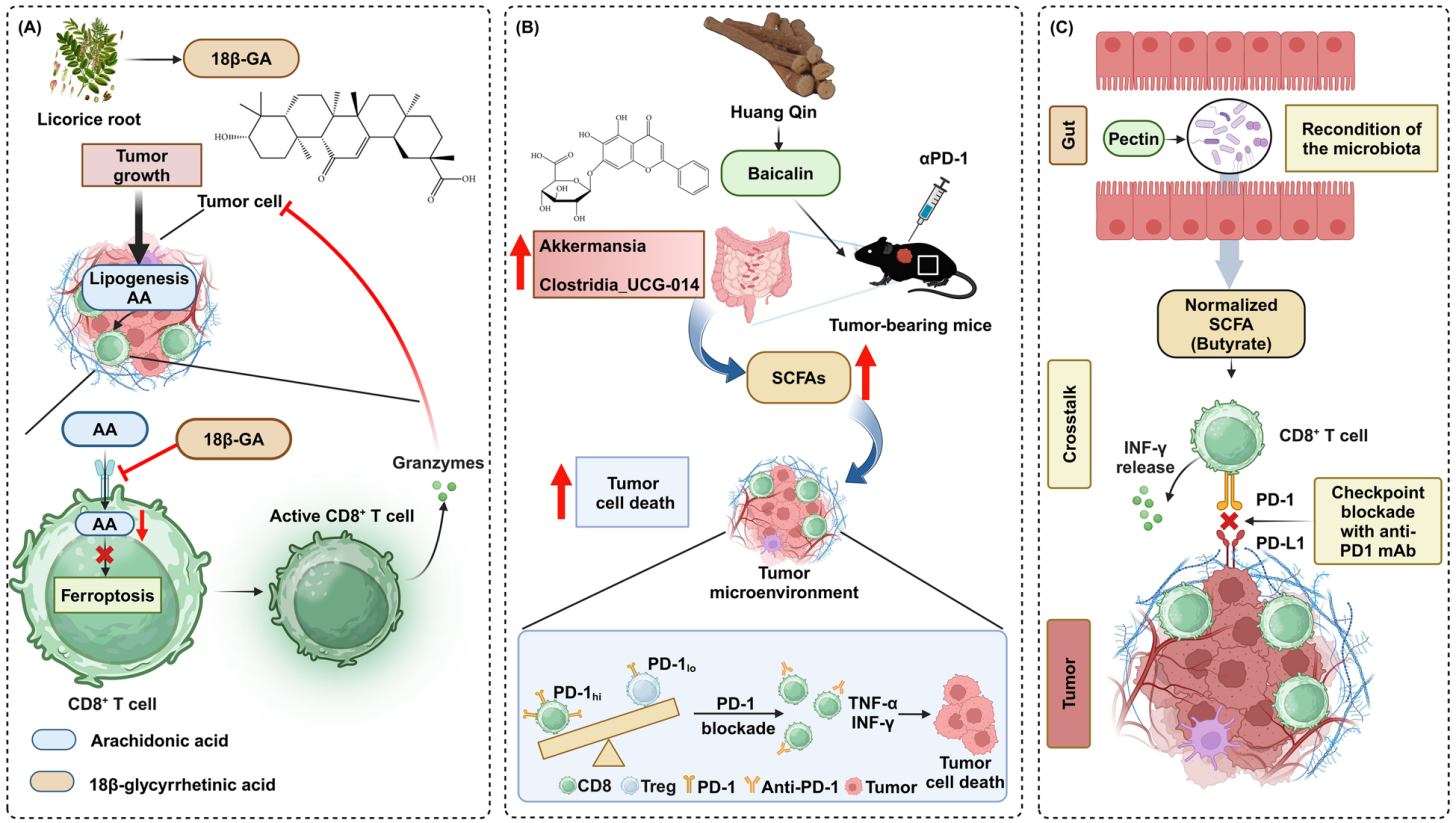
Exogenous plant-derived metabolites for enhancing CD8^+^ T cell-based immunotherapy. **A** Licorice root-derived 18β-glycyrrhetinic acid (18β-GA) inhibits tumor growth by inhibiting ferroptosis in CD8^+^ T cells through the modulation of arachidonic acid metabolism and increasing the release of granzymes. **B** Huang Qin-derived baicalin promotes SCFA production in the gut microbiota, particularly increasing bacteria Akkermansia and Clostridia_UCG-014, which modulates the TME and leads to tumor cell death. This effect is further amplified by PD-1 blockade in tumor-bearing mice, improving CD8^+^ T cell-mediated tumor killing. (C) Pectin reconditions the gut microbiota, normalizing SCFA levels, particularly butyrate, which enhances CD8^+^ T cell function through increased IFN-γ release and improves the efficacy of checkpoint blockade with anti-PD1 monoclonal antibodies. These plant-derived metabolites collectively improve CD8^+^ T cell-based immunotherapy by modulating the TME and enhancing immune cell function. Created in BioRender.com

Plant-derived metabolites play a significant role in enhancing CD8^+^ T cell-based immunotherapy, offering novel avenues for cancer treatment. These natural compounds, isolated from various plants, possess unique properties that can modulate immune responses effectively (Fig. 6; Table 2). For instance, 18β-glycyrrhetinic acid (18β-GA), a metabolite of glycyrrhizic acid from licorice root, enhances CD8^+^ T cell function and inhibits their ferroptosis in LC models. This immunomodulatory action is achieved by 18β-GA's suppression of CD36 expression, which disrupts arachidonic acid-mediated pathways involved in ferroptosis [109]. Additionally, genistein, a soy-derived isoflavone, enhances host resistance to B16F10 tumors in female B6C3F1 mice by modulating immune responses. Treatment with genistein significantly increased cytotoxic T-cell activity in a dose-dependent manner and elevated IL-2-stimulated natural killer cell (NK cell) activity at higher doses. Although serum genistein and its metabolites did not directly inhibit B16F10 cell proliferation, their role in boosting CD8^+^ T cell and NK cell activities suggests potential therapeutic avenues for CD8^+^ T cell-based immunotherapy [110]. Moreover, freeze-dried black raspberries, their anthocyanin fraction, and the metabolite protocatechuic acid effectively modulate immune responses in N-nitrosomethylbenzylamine (NMBA)-treated rats, altering cytokine expression and reducing innate immune cell infiltration in the esophagus. These components significantly decrease proinflammatory IL-1β levels and increase anti-inflammatory IL-10, along with enhancing IL-12 expression. This upregulation of IL-12 is pivotal as it activates cytolytic natural killer and CD8^+^ T cells, contributing to the inhibition of esophageal tumorigenesis through improved immunotherapy mechanisms [111].

**Table 2:** Exogenous plant-derived metabolites and human endogenous metabolites in disease: CD8^+^ T cell modulation

Metabolites	Associated disease	Mechanism of action	Specific CD8^+^ T cell effects	Clinical application	Refs.
18β-GA	LC	Suppress CD36 expression, disrupts arachidonic acid-mediated pathways	Enhance CD8^+^ T cell function and inhibit ferroptosis	Enhance CD8^+^ T cell efficacy in LC immunotherapy	[109]
Genistein	Melanoma	Increase cytotoxic T-cell activity and elevate IL-2-stimulated NK cell activity	Enhance CD8^+^ T cell activity	Enhance CD8^+^ T cell efficacy in melanoma immunotherapy	[110]
Protocatechuic acid	Esophageal cancer	Decrease IL-1β levels and enhance IL-10 and IL-12 expression	Enhance CD8^+^ T cell function	Enhance CD8^+^ T cell efficacy in esophageal cancer immunotherapy	[111]
Baicalin	NSCLC	Modulate gut microbiota and increase SCFA production	Boost IFN-γ^+^ and TNF-α^+^ CD8^+^ T cell levels	Augment anti-PD1 therapy effectiveness in NSCLC	[112]
Pectin	CRC	Modulate gut microbiota and increase butyrate production	Enhance infiltration	Augment anti-PD1 therapy effectiveness in CRC	[113]
Glutarate	Melanoma, OV, Cervical cancer, Lymphoma	Inhibit αKGDDs via glutarylation of PDH E2	Enhance cytotoxicity and boost abundance	Potential role in the improvement of immunotherapy	[114]
Lactate	CRC	Inhibit HDAC, boost H3K27 acetylation, upregulate TCF7 expression	Increase stemness in CD8^+^ T cells	Potential as an immunotherapeutic agent	[115]
2-ME	Melanoma	Boost CD8^+^ T cell infiltration and upregulate PD-L1 on tumor cells	Enhance anti-PD1 therapy	Augment anti-PD1 therapy effectiveness in melanoma	[116]
Pantothenate	Melanoma	Enhance CoA production, boost OXPHOS and the Tc22 phenotype	Boost Tc22 phenotype	Augment anti-PD1 therapy effectiveness in melanoma	[117]
atRA	Melanoma	Enhance CD8^+^ T cell responses, upregulate MHC class I expression	Increase differentiation and cytotoxicity	Enhance melanoma immunotherapy efficacy	[118]

Notably, plant-derived compounds or metabolites might indirectly modulate the immune response by regulating the host microbiota. Baicalin enhances anti-PD-1 immunotherapy by modulating gut microbiota and increasing SCFA production in mice. This modulation results in a favorable shift in the PD-1^+^ CD8^+^ T cell to Treg ratio and boosts IFN-γ^+^ and TNF-α^+^ CD8^+^ T cell levels within the TME [112]. Additionally, pectin supplementation significantly enhances anti-PD-1 monoclonal antibody efficacy in CRC by modulating gut microbiota and increasing butyrate production. In tumor-bearing mice humanized with CRC patient microbiota, pectin improved T cell infiltration and activation within tumors. This effect is mediated by butyrate, which directly promotes T cell responses [113].

## Supplementing human endogenous metabolites to enhance CD8^+^ T cell-based immunotherapy

**Fig. 7:**
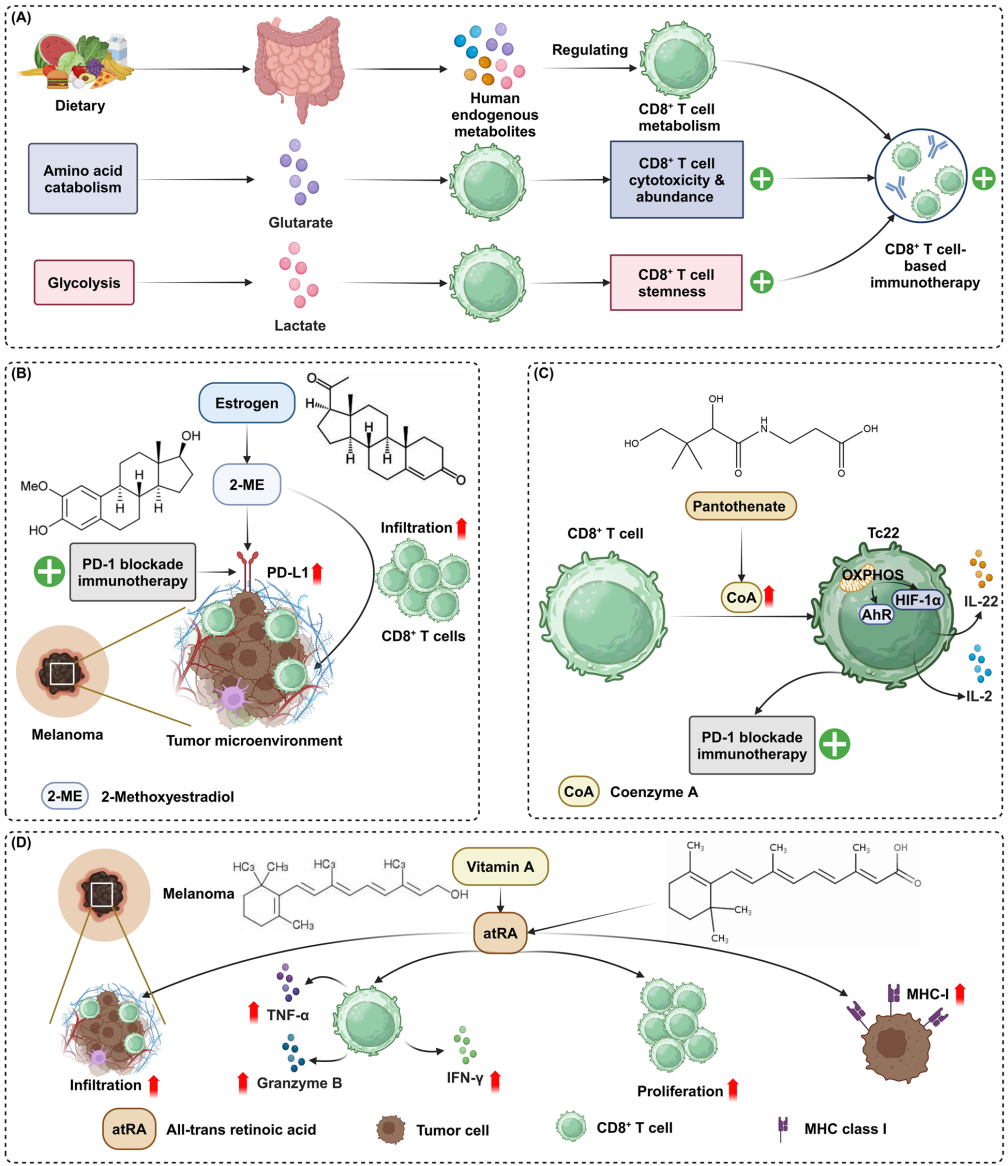
Enhancing CD8^+^ T cell immunotherapy with human endogenous metabolites. **A** Dietary intake influences the production of human endogenous metabolites such as glutarate and lactate, which regulate CD8^+^ T cells metabolism, enhancing their cytotoxicity, abundance, and stemness. This, in turn, improves CD8^+^ T cell-based immunotherapy. **B** 2-ME, derived from estrogen, increases PD-L1 expression and CD8^+^ T cell infiltration in the TME, boosting the efficacy of PD-1 blockade immunotherapy in melanoma. **C** Pantothenate enhances Coenzyme A production in CD8^+^ T cells, activating OXPHOS and upregulating HIF-1α, which promotes the Tc22 phenotype and improves PD-1 blockade immunotherapy outcomes. **D** atRA, a derivative of vitamin A, increases TNF-α, granzyme B production and IFN-γ production, and promotes CD8^+^ T cell proliferation, while also upregulating MHC class I expression on tumor cells. This combination enhances CD8^+^ T cell infiltration and anti-tumor activity in melanoma. Created in BioRender.com

The efficacy of CD8^+^ T cell-based immunotherapy is profoundly influenced by cellular metabolism, which governs the energy supply and biosynthetic demands of these crucial immune cells [79, 119]. Supplementing human endogenous metabolites represents a strategic approach to modulate these metabolic pathways, directly impacting CD8^+^ T cell efficacy (Fig. 7).

Glutarate, a metabolite from amino acid catabolism, modulates T cell function and differentiation by inhibiting α-ketoglutarate-dependent dioxygenases and through glutarylation of the pyruvate dehydrogenase E2 subunit (PDH E2). The administration of diethyl glutarate enhances CD8^+^ T cell cytotoxicity and increases their abundance both peripherally and within tumors [114]. Additionally, lactate, derived from glycolysis, enhances antitumor immunity by increasing stemness in CD8^+^ T cells through the inhibition of histone deacetylase activity. This action boosts H3K27 acetylation at the transcription factor 7 (TCF7) super enhancer, upregulating TCF7 expression and promoting a stem-like phenotype. Sodium lactate administration in tumor-bearing mice shows significant CD8^+^ T cell-dependent tumor growth inhibition, highlighting lactate's potential as an immunotherapeutic agent [115]. Notably, 2-Methoxyestradiol (2-ME) enhances the infiltration of cytotoxic CD8^+^ T cells into the TME and increases PD-L1 expression on tumor cells. These effects suggest that 2-ME could effectively augment PD-1 blockade immunotherapy, positioning it as a potential synergistic agent for treating melanoma [116]. Supplementation with pantothenate enhances Coenzyme A (CoA) production, boosting OXPHOS and the type 22 cytotoxic T lymphocyte (Tc22) phenotype, which in turn augments the effectiveness of anti-PD-L1 therapy in murine tumor models and correlates with improved responses to anti-PD1 therapy in melanoma patients [117]. Interestingly, topical application of all-trans retinoic acid (atRA) effectively suppresses melanoma growth by enhancing CD8^+^ T cell responses. atRA treatment increases the differentiation and cytotoxic functions of effector CD8^+^ T cells, evidenced by elevated levels of granzyme B, TNF-α, IFN-γ, and proliferation markers. atRA's tumor-inhibitory effects are CD8^+^ T-cell dependent and are accompanied by upregulated MHC class I expression, enhancing the killing efficacy of these cells [118].

## Challenges and future perspectives

Normal metabolic processes are fundamental to the optimal functioning of CD8^+^ T cells, as they govern vital aspects such as energy production, biosynthesis, and redox balance, which are essential for their activation, proliferation, and survival. Given this critical dependency, harnessing exogenous metabolites emerges as a promising strategy to modulate these metabolic pathways, potentially enhancing the efficacy of CD8^+^ T cell-based immunotherapy. While the therapeutic augmentation of T cell metabolism with exogenous agents offers considerable promise, it also presents significant challenges.

Firstly, the precise control of metabolic modulation in CD8^+^ T cell-based immunotherapy is paramount to optimize therapeutic outcomes while minimizing adverse effects [120]. This control is critical because metabolic pathways are inherently complex and interconnected, with potential for broad systemic impacts beyond the targeted therapeutic effects. Effective metabolic modulation in CD8^+^ T cell therapy requires precise targeting, dosing, and timing. Selective targeting of metabolic pathways that are active or dysregulated within CD8^+^ T cells in the TME is crucial to enhance T cell function without affecting non-target cells, thereby reducing off-target effects and systemic toxicity. Dose optimization is essential to avoid overstimulation that could lead to metabolic exhaustion or excessive production of ROS, potentially compromising cell integrity. Additionally, the timing of metabolite administration should be strategically planned to coincide with specific phases of T cell activation and function, with certain metabolic boosters being more beneficial during the initial activation phase to prevent metabolic burnout and ensure long-term cell viability and functionality.

Secondly, developing targeted delivery systems that can specifically deliver metabolites to CD8^+^ T cells in the TME is crucial. Engineering extracellular vesicles (EVs) from probiotics or utilizing nanosystems to enrich or carry specific metabolites presents an innovative strategy to enhance CD8^+^ T cell-based immunotherapy [121]. For instance, EVs from *Lactobacillus rhamnosus GG* (LGG-EV) enhance the efficacy of anti-PD-1 immunotherapy in CRC by modulating intestinal immunity. Optimized production of LGG-EV increases the CD8^+^ T/CD4^+^ T cell ratio and MHC class II^+^ dendritic cell presence in tumor tissues [122]. By harnessing the intrinsic biocompatibility and targeting capabilities of EVs, these engineered particles can be designed to deliver immunomodulatory metabolites directly to the TME. This approach not only ensures the localized concentration of therapeutic agents but also reduces systemic side effects. Upon delivery, these metabolites could alter the metabolic landscape of the tumor, promoting the activation and proliferation of cytotoxic T cells. Additionally, the use of nanosystems allows for the precise control over the release kinetics of the loaded metabolites, optimizing their availability during critical phases of the immune response. Nanoliposome-loaded C6-ceramide (LipC6) enhances immunotherapy efficacy in liver tumor-bearing mice by modulating TAMs and boosting CD8^+^ T cell activity. LipC6 administration reduces TAM numbers, induces M1 phenotype differentiation, lowers ROS production, and decreases Protein kinase B (AKT)-mediated tumor cell proliferation while increasing apoptosis [123]. Such targeted delivery systems could significantly amplify the efficacy of CD8^+^ T cell therapies, providing a potentiated response against malignancies with previously limited treatment options.

Thirdly, exploring traditional Chinese medicine, conventional chemotherapeutic agents, and plant-derived bioactive compounds offers a promising strategy to enhance immunotherapy outcomes. These bioactive compounds can modulate the gut microbiota or be metabolized into beneficial metabolites, thereby indirectly enhancing the efficacy of immune-based treatments. By influencing the composition and function of the gut microbiota, these compounds may foster an immunological milieu conducive to effective CD8^+^ T cell responses. For instance, astragalus polysaccharides enhance CD8^+^ T cell-mediated tumor suppression in melanoma by modulating the gut microbiota, thus reducing myeloid-derived suppressor cells and associated immunosuppressive factors like Arginase 1, IL-10, and TGF-β. These changes correlate with increased levels of metabolites such as glutamate and creatine, critical for inhibiting tumor growth [124]. Additionally, SN-38, an active metabolite of irinotecan, modulates tumor immunity by enhancing FoxO3a expression and suppressing c-Myc and PD-L1 in cancer cells. This metabolic intervention promotes interferon-γ secretion and cytotoxic activity of natural killer cells. Combining SN-38 with anti-PD-1 therapy significantly boosts CD8^+^ T-cell and NK cell infiltration in tumor tissues, effectively suppressing tumor growth in murine models [125].

Lastly, the inherent variability in metabolic states among patients underscores the necessity for personalized approaches in CD8^+^ T cell-based immunotherapy using exogenous metabolites. Tailoring treatment to individual metabolic profiles through comprehensive metabolic profiling can optimize the efficacy of metabolite interventions [126]. Incorporating real-time metabolic monitoring and leveraging predictive computational models can further enhance treatment customization, allowing for dynamic adjustments in response to changes in the TME and individual patient condition, thus maximizing therapeutic outcomes while minimizing adverse effects.

## Conclusions

In conclusion, the targeted modulation of the metabolic environment of CD8^+^ T cells represents a significant advancement in enhancing the effectiveness of current immunotherapies. The strategic application of exogenous metabolites, supported by robust in vivo evidence, has emerged as an effective strategy to optimize the metabolic functions of these pivotal immune cells, thereby improving their cytotoxic capabilities and longevity. This innovative approach not only addresses the limitations of traditional therapies but also offers distinct advantages by increasing the precision and adaptability of immune responses.

Traditional immunotherapeutic strategies often overlook the metabolic demands of CD8^+^ T cells, which can limit their efficacy and sustainability. Integrating metabolic interventions extends the functional lifespan and enhances the potency of these cells in combating cancer and infectious diseases. However, realizing the full potential of this strategy involves addressing challenges such as precise metabolic control, the development of targeted delivery systems, and the customization of treatments for individual metabolic diversity. Future research should aim to systematically integrate these metabolic strategies in clinical trials to evaluate their efficacy and safety profiles. By emphasizing the advantages of metabolic interventions over traditional methods, the importance and timeliness of this innovative approach are underscored. As this field evolves, it is poised to make substantial contributions to the advancement of cancer treatment and the broader application of immunotherapies, representing a major breakthrough in harnessing the full capabilities of CD8^+^ T cell-based therapies.

## Data Availability

No datasets were generated or analysed during the current study.

## Abbreviations

18β-GA: 18β-Glycyrrhetinic acid
2-ME: 2-Methoxyestradiol
αKGDDs: αKG-dependent dioxygenases
AhR: Aryl hydrocarbon receptor
AIH: Autoimmune hepatitis
AKT: Protein kinase B
AML: Acute myeloid leukemia
ApoA1: Apolipoprotein A1
ATF4: Activating transcription factor 4
ATM: Ataxia-telangiectasia mutated
atRA: All-trans retinoic acid
BC: Breast cancer
BCAA: Branched-chain amino acid
CAR: Chimeric antigen receptor
CCL20: C–C motif chemokine ligand 20
CHOP: C/EBP homologous protein
CoA: Coenzyme A
CRC: Colorectal cancer
CTLA-4: Cytotoxic T-lymphocyte-associated protein 4
D-2-HG: D-2-hydroxyglutarate
DC: Dendritic cell
ER: Endoplasmic reticulum
ERK1/2: Extracellular regulated protein kinases 1/2
EV: Extracellular vesicle
FAO: Fatty acid oxidation
FAS: Fatty acid synthesis
FFAR3: Free fatty acid receptor 3
GPR41: G-protein-coupled receptor 41
GPR43: G-protein-coupled receptor 43
HDAC: Histone deacetylase
H3K27: Histone 3 lysine 27I3A
I3A: Indole-3-aldehyde
ICB: Immune checkpoint blockade
ICI: Immune checkpoint inhibitor
ID2: Inhibitor of DNA binding 2
IDH: Isocitrate dehydrogenase
IDO: Indoleamine 2,3-dioxygenase
IEL: Intraepithelial lymphocyte
IL: Interleukin
iME: Isomycousnine enamine
ITE: 2-(1′H-indole-3′-carbonyl)-thiazole-4-carboxylic acid methyl ester
LA: Linoleic acid
LC: Lung cancer
LGG-EV: Extracellular vesicles from *Lactobacillus rhamnosus GG*
LipC6: Nanoliposome-loaded C6-ceramide
MAPK: Mitogen-activated protein kinase
MHC class I: Major histocompatibility complex class I
MHC class II: Major histocompatibility complex class II
MPEC: Memory precursor effector cell
mTOR: Mammalian target of the rapamycin
mTORC1: Mechanistic target of rapamycin complex 1
NaB: Sodium butyrate
NK cell: Natural killer cell
NMBA: N-nitrosomethylbenzylamine
NSCLC: Non-small cell lung cancer
OPC: Oropharyngeal cancer
OXPHOS: Oxidative phosphorylation
PD-1: Programmed death receptor 1
PD-L1: Programmed cell death ligand 1
PDH E2: Pyruvate dehydrogenase E2 subunit
PERK: Protein kinase R-like endoplasmic reticulum kinase
PI3K: Phosphatidylinositol 3-kinase
PTEN: Phosphatase and tensin homolog
ROS: Reactive oxygen species
S-2-HG: S-2-hydroxyglutarate
SAA3: Serum amyloid protein A 3
SCFA: Short-chain fatty acid
SENP7: SUMO-specific protease 7
SLC6A8: Solute carrier family 6 member 8
SLEC: Short-lived effector cell
SOD2: Superoxide dismutase 2
STAT1: Signal transducer and activator of transcription 1
STAT3: Signal transducer and activator of transcription 3
TAM: Tumor-associated macrophage
Tc1: Type 1 cytotoxic T lymphocyte
Tc17: Type 17 cytotoxic T lymphocyte^+^
Tc22: Type 22 cytotoxic T lymphocyte^+^
TCF7: Transcription factor 7
T_CM_: Central memory T cell
TCR: T cell receptor
TD-139: Olitigaltin
T_EM_: Effector memory T cell
Tet2: Ten-eleven translocation 2
TIL: Tumor-infiltrating T cell
TLR4: Toll-like receptor 4
TLR5: Toll-like receptor 5
TMAO: Trimethylamine N-oxide
TME: Tumor microenvironment
TNBC: Triple-negative breast cancer
TNF-α: Tumor necrosis factor-α
Treg: Regulatory T cell
T_RM_: Tissue-resident memory T cell
USP21: Ubiquitin specific peptidase 21
